# Alternative splicing in endothelial cells: novel therapeutic opportunities in cancer angiogenesis

**DOI:** 10.1186/s13046-020-01753-1

**Published:** 2020-12-07

**Authors:** Anna Di Matteo, Elisa Belloni, Davide Pradella, Ambra Cappelletto, Nina Volf, Serena Zacchigna, Claudia Ghigna

**Affiliations:** 1 Istituto di Genetica Molecolare, “Luigi Luca Cavalli-Sforza”, Consiglio Nazionale delle Ricerche, via Abbiategrasso 207, 27100 Pavia, Italy; 2 Cardiovascular Biology Laboratory, International Centre for Genetic Engineering and Biotechnology (ICGEB), 34149 Trieste, Italy; 3 Department of Medical, Surgical and Health Sciences, University of Trieste, 34149 Trieste, Italy

**Keywords:** Alternative splicing; RNA binding proteins, Endothelial cells, Angiogenesis, Vascular biology, Anti-angiogenic therapy

## Abstract

Alternative splicing (AS) is a pervasive molecular process generating multiple protein isoforms, from a single gene. It plays fundamental roles during development, differentiation and maintenance of tissue homeostasis, while aberrant AS is considered a hallmark of multiple diseases, including cancer. Cancer-restricted AS isoforms represent either predictive biomarkers for diagnosis/prognosis or targets for anti-cancer therapies. Here, we discuss the contribution of AS regulation in cancer angiogenesis, a complex process supporting disease development and progression. We consider AS programs acting in a specific and non-redundant manner to influence morphological and functional changes involved in cancer angiogenesis. In particular, we describe relevant AS variants or splicing regulators controlling either secreted or membrane-bound angiogenic factors, which may represent attractive targets for therapeutic interventions in human cancer.

**Supplementary Information:**

The online version contains supplementary material available at 10.1186/s13046-020-01753-1.

## Background

### Introduction: from the theory of angiogenesis to an orchestra of alternatively spliced angiogenic genes

In the 1970s Judah Folkman revolutionized the field of angiogenesis with his radical idea that tumor growth could be halted by depriving it of blood supply. It all started as a by-product of an investigation originally designed to test the efficacy of haemoglobin-plasma solution as a blood substitute for prolonged extracorporeal perfusion. Folkman was testing whether haemoglobin-plasma solution sustained viability of dog thyroid glands ex vivo. To prove tissue viability, he implanted mouse tumor cells into dog glands and observed that the neoplastic mass stopped growing after having reached a modest size, but grew rapidly again if transplanted back into a living mouse. He also noticed that retro-transplanted tumors were decorated by a network of tiny blood vessels, which were not present in tumors grown inside the thyroid glands [1]. Later, experiments in the hamster cheek pouch showed that capillary sprouts grew even if tumor cells were separated from the host stroma by a porous filter, suggesting the existence of an active humoral factor capable of driving tumor neovascularization (also named angiogenesis) [2, 3]. This factor was isolated by Folkman and initially named tumor-angiogenesis factor, TAF. It could be purified from human and animal tumors, as well as from the placenta, and showed remarkable mitogenic activity toward endothelial cells (ECs) in multiple assays [2–4]. This was the first evidence that tumor growth is always accompanied by new blood vessel formation and paved the way to the idea of blocking angiogenesis to halt tumor growth. In its original assumption, the concept of anti-angiogenesis would prevent new vessel sprouts from penetrating into an early tumor and keep it in an avascular and dormant state, in which it cannot exceed 2–3 mm size [5]. While this concept was initially widely criticized, its potential efficacy in treating cancer started to emerge a few years later, when Folkman teamed up with his students and monitored the grow of cancer cells when implanted into either the avascular anterior chamber of the eye or the iris, which contains abundant blood vessels. Avascular implants in the anterior chamber barely grew and soon became dormant. In contrast, the same tumors grew 4000-fold faster in the vascularized iris. This clearly demonstrated that tumor growth depends on blood supply and tumor dormancy is caused by lack of vascularization and not by cell cycle arrest or immune control, as previously believed [6]. Discovery of TAF triggered the search for numerous angiogenic molecules, including vascular endothelial growth factor (VEGF), fibroblast growth factor (FGF), angiogenin, and many others [7].

Over the years, a more complex situation has emerged, and the original names (i.e. VEGF or FGF) are currently used to indicate families of proteins, each one existing in multiple splicing isoforms. It has also become clear that members of the same family, but also alternative splicing (AS) variants of the same protein, can elicit either pro- or anti-angiogenic activities. Their relative abundance in cancer significantly contributes to the effective formation of new blood vessels and thus AS represents an attractive target for the development of innovative therapies.

### Alternative splicing

**Fig. 1:**
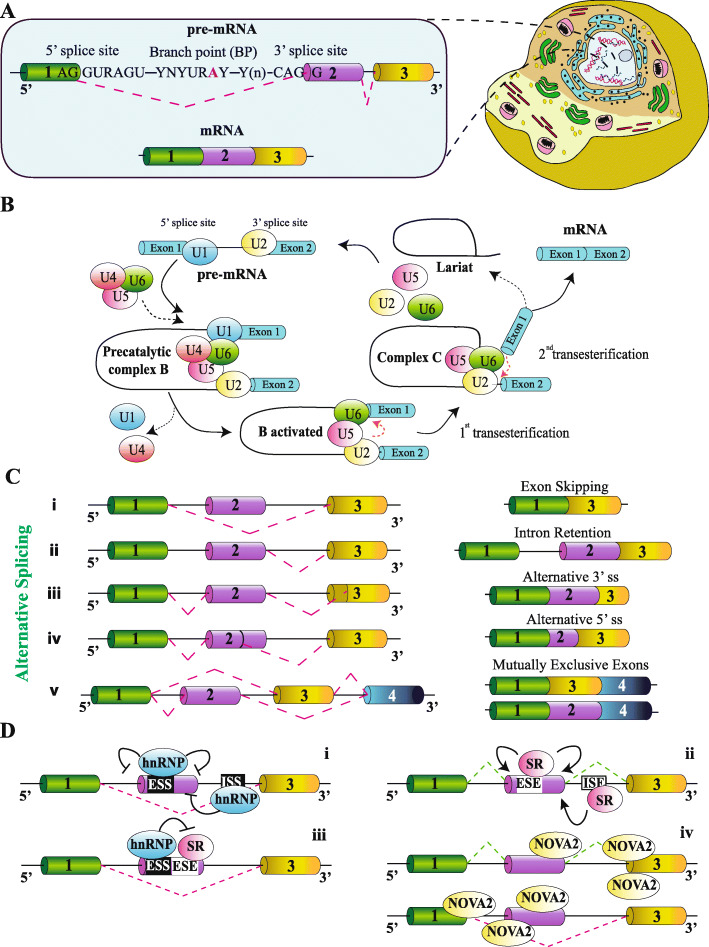
Splicing reaction and its regulation. **a**) Splicing, which occurs in the nucleus of eukaryotic cells, required cis-acting sequences located in the pre-mRNA at the exon/intron and intron/exon junctions: the 5' splice site, the branch point or BP, the polypyrimidine Y tract and 3' splice site. R=purine; N=any nucleotide; Y=pyrimidine. **b**) Splicing involved two consecutive transesterification reactions carried out by the spliceosomal machinery, which is composed by five small nuclear ribonucleoproteins (U1, U2, U4, U6, and U5 snRNPs). The different complexes formed by snRNPs, pre-mRNA and a large number of proteins (not indicated) are depicted. The final product of the splicing reaction is the mature mRNA in which exons are ligated together, whereas intron is released in the form of a looped structure (the lariat). Thin black lines=introns; blue cylinders=exons. **c**) Different types of AS reaction: (i) exon skipping; (ii) intron retention; (iii) alternative 3' splice sites (ss); (iv) alternative 5' splice sites (ss); (v) mutually exclusive exons. **d**) AS regulation requires the combined action of trans- and cis-acting elements. (i) Generally, hnRNPs by binding intronic or exonic splicing silencers (ISS or ESS) directly prevent the recognition of the regulated exon by the spliceosomal machinery (red dashed lines). (ii) On the contrary, exonic or intronic splicing enhancers (ESE or ISE) are bound by SR factors able to stimulate spliceosome assembly on 5' and 3' splice sites (blue dashed lines). (iii) hnRNPs can also polymerize along the exon and displace ESE-bound SR factors, thus preventing exon recognition. (iv) Differently, other SRFs (like NOVA2) are able to promote or repress exon recognition depending on the location of their binding sites on the pre-mRNA. For example, NOVA2 stimulates exon skipping (red dashed lines) when bound to exonic or upstream intronic YCAY (Y=pyrimidine) clusters, while it promotes exon inclusion (green dashed lines) when associated to downstream intronic motifs

In eukaryotic cells, intron removal from primary transcripts (pre-mRNAs) by splicing is an obligatory step before mature mRNAs are transported into the cytoplasm for their translation (Fig. 1a). Splicing is realized in the nucleus by a complex and dynamic molecular machinery, the spliceosome, which recognizes short consensus motifs close to the exon-intron and intron-exon junctions: the 5′ and the 3′ splices sites, the branch point, and the polypyrimidine tract [8] (Fig. 1a). These sequences are bound by spliceosome components (such as snRNPU1, snRNPU2, SF1, U2AF65, and U2AF35), which undergo multiple conformational rearrangements, leading to splicing catalysis (Fig. 1b).

While in constitutive splicing an exon is always included in the mature mRNA, AS is characterized by intron retention, exon skipping, usage of alternative 5′ or 3′ splice sites, and mutually exclusive exons (Fig. 1c). In this way, AS generates multiple mRNAs and, as a consequence, different proteins with diverse structure, function, stability, and sub-cellular localization [9]. AS correlates with organism complexity, affecting 95% of human protein-coding genes [10, 11] and only 25–60% of *Drosophila melanogaster* and *Caenorhabditis elegans* genes, respectively [12–14].

Alternatively spliced mRNAs frequently display a tissue-specific expression [11] and encode for specialized proteins involved in development, differentiation and maintenance of tissue homeostasis [15]. AS often affects domains involved in protein-protein interaction, suggesting its crucial role in controlling connected signaling cascades [15].

Splicing signals (for example 3′ splice sites) are often short and degenerated. The intrinsic weakness of these motifs determines their low affinity for spliceosome components. This, in combination with auxiliary sequences that are located either within exons or in the adjacent introns, creates the opportunity to realize AS schemes. Auxiliary splicing signals are recognized by RNA binding proteins (RBPs), which either stimulate (enhancers) or inhibit (silencers) spliceosome assembly on the pre-mRNA [16] (Fig. 1d). The majority of the splicing enhancers are purine-rich motifs and are bound by Serine-Arginine-rich (SR) proteins [17]. On the contrary, splicing silencers are diverse in sequence and they are mainly bound by heterogeneous nuclear ribonucleoproteins (hnRNPs) [18]. Similar to transcription regulatory sequences, splicing enhancers and silencers are often clustered on the pre-mRNA. Consequently, several SR proteins and hnRNPs act in either synergistic or antagonistic manner. For example, SR proteins can block the binding of hnRNPs to a nearby silencer sequence and thus inhibit their negative effect on splicing (Fig. 1d). Therefore, the relative levels of SR proteins and hnRNPs determine the outcome of the AS reaction. While SR proteins are ubiquitously expressed, a few splicing regulatory factors (SRFs) display a more restricted pattern of expression, thus contributing to tissue-specific gene expression programs [15]. Finally, reading of the “splicing code” depends on multiple elements that can mask splicing signals, including secondary structures in the pre-mRNA [19], chromatin organization, epigenetic modifications [20], and RNA pol II elongation rate [21].

AS dysregulation has emerged as an important genetic modifier in tumorigenesis [22]. Mutations in splicing sequences and/or altered expression of SRFs are frequent in tumors [23]. A number of SRFs behave as *bona fide* oncogenes [24, 25], whereas others act as tumor suppressors [26, 27]. Since a specific SRF controls hundreds (if not thousands) of target genes, its aberrant expression in cancer cells results in global changes of AS signatures, potentially driving either oncogene activation or inhibition of tumor suppressors [22, 28]. Transcriptome sequencing data from clinical samples indicate that several AS errors are cancer-restricted and particularly relevant for the diagnosis, prognosis and targeted therapy of multiple cancer types [29, 30].

## Main text

### Genome-wide AS changes in ECs

Genome-wide studies have revealed that AS acts in a specific and non-redundant manner to influence EC response to diverse stimuli [31, 32]. For example, blood flow determines different levels of shear stress in ECs depending on the anatomical site, as well as on pathological conditions (i.e. atherosclerosis, aneurysms) [33, 34]. ECs sense and convert this mechanical stimulus into an intracellular response through mechanosensor receptors expressed on EC surface. A paradigmatic example of AS regulation by shear stress refers to specific isoforms of the extracellular matrix (ECM) protein fibronectin (EDA-FN and EDB-FN), which are expressed in pathological conditions, but absent in the normal quiescent vasculature [35], as discussed later. More recent RNA-seq analysis further demonstrated a more extensive role of AS in endothelial response to altered hemodynamics, which affects multiple factors implicated in vascular remodeling, such as PECAM1, YAP1, and NEMO [31].

Another important stimulus able to globally remodel EC transcriptome is hypoxia, a condition in which cells are deprived of oxygen, as happens in the center of a tumor mass [36]. Both tumor and stromal cells release pro-angiogenic factors that stimulate the formation of immature, disorganized, and leaky vessels [37], further enhancing the hypoxic condition of the tumor microenvironment [38]. The *hypoxia inducible transcription factors* HIF-1 and HIF-2 activate a gene expression program required for EC adaptation to insufficient oxygen supply [39]. Since HIF-1 and HIF-2 act as transcription factors, previous transcriptome analyses of hypoxic ECs have been mainly focused on changes in mRNA steady-state levels and proteomic profiling [36, 40], whereas very few studies have investigated the global impact of AS regulation during oxygen deprivation. Splicing-sensitive microarrays applied to human umbilical venous ECs (HUVECs) exposed to hypoxic conditions identified genome-wide AS changes [41, 42], affecting factors involved in cytoskeleton organization (*CASK*, *ITSN1*, *SPTAN1*, and *TPM1),* cell adhesion (*NRP1* and *ROBO1*), apoptosis (*LARP6*) and universal regulators of gene expression (*SH3KBP*, *RPP9*, *ZNF589*, *HMGA2, CELF1,* and *MAX*). These initial studies used microarrays, which are limited in the number and type of AS events that could be detected [43]. RNA-seq approaches have more recently allowed the identification of robust hypoxia-induced AS programs in cancer cells [44, 45], although detailed AS signatures in hypoxic ECs are still missing and will require further investigations.

### AS isoforms acting on the extracellular space during physiological and tumor angiogenesis

**Fig. 2:**
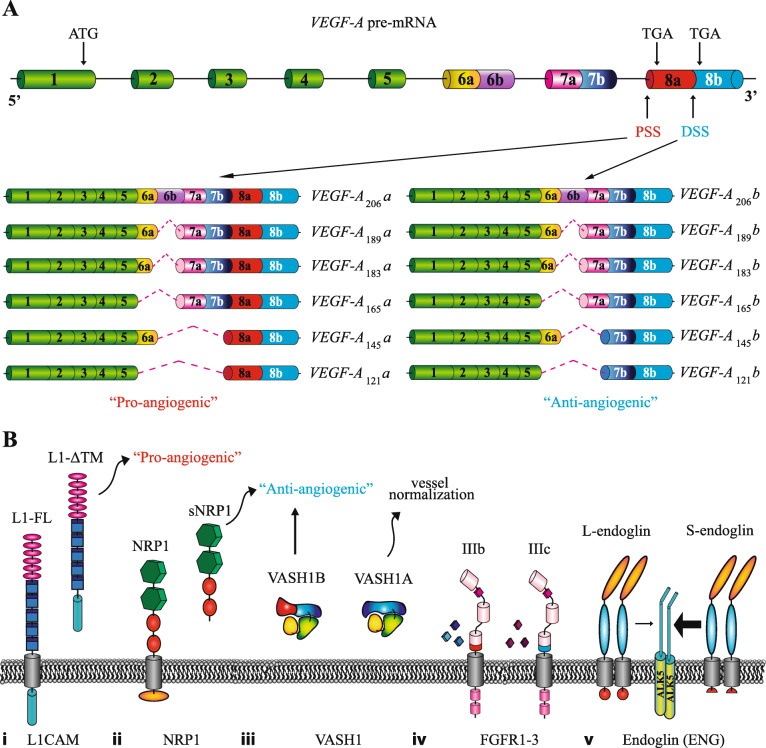
Alternative splicing in genes with important role in angiogenesis. **a**) Schematic representation of VEGF-A alternative splicing isoforms. *VEGF-A* gene with constitutive (green) and alternative (other colours) exons is shown. Thin black lines=introns. PSS: proximal splice site; DSS: distal splice site. Depending on the recognition of different 3' splice sites (PSS or DSS) in exon 8, two classes of VEGF-A isoforms with opposite role in angiogenesis − “pro-angiogenic” (VEGF-Axxxa) or “anti-angiogenic” (VEGF-Axxxb) − are generated. In addition, inclusion/exclusion of alternative exons 6 and 7 give rise to isoforms with different length and heparin affinity. **b**) Other examples of genes regulated by AS with role in angiogenesis. From the left: (i) L1CAM: skipping of the exon encoding for TM domain (grey cylinder) generates a soluble isoform (L1-ΔTM) with pro-angiogenic functions; (ii) soluble NRP1 isoforms (sNRP1: s_11_NRP1, s_12_NRP1, s_III_NRP1, s_IV_NRP1) that lack the TM domain and the cytoplasmic tail (grey and orange cylinders) act as decoy receptors for NRP1 ligands and show “anti-angiogenic” properties; (iii) whereas the VASH1A isoform is able to promote vessels normalization, the VASH1B protein (with a diverse C-terminal region involved in heparin binding), has an “anti-angiogenic” activity; (iv) mutually exclusive usage of exon 8 or 9 in FGFR1-3 pre-mRNAs gives rise to distinct isoforms (IIIb and IIIc) that differ for the last portion of the immunoglobulin-like domain 3 (IgIII, indicated with red or blue cylinders) and their ligand specificity; (v) Short endoglin (S-endoglin) has a short cytoplasmic tail (red circle) compared to the long (L-endoglin) isoform. As result S-endoglin and L-endoglin shown a different ability to interact with the TGFβ type I receptor ALK5. Small arrow= low interaction; Big arrow= strong interaction. The different protein domains are indicated by coloured geometric forms. TM = transmembrane domain

Numerous proteins generated by AS affect EC biology. Here, we focus on events affecting proteins that are either membrane-bound or secreted, and thus represent putative targets for anti-angiogenic therapy (summarized in Table 1 and Fig. 2). A more exhaustive list of AS isoforms potentially modulating cancer angiogenesis is provided in Supplementary Table 1 (Additional files 1 and 2).

**Table 1:** Alternatively spliced isoforms of angiogenesis-related genes and their potential use for anti-angiogenic therapy

GENE	AS variant	Relevance in cancer angiogenesis
*VEGF-A*	VEGF-Axxxa	**Expression/function** Overexpressed by a wide variety of human tumors. Pro-angiogenic function, produced by both cancer cells and ECs [46].
	VEGF-Axxxb	**Expression/function** Anti-angiogenic function, generally downregulated in cancer [46]; not detected in normal or tumor ECs [47]. **Examples of potential use for therapy** SRPK1 inhibitors to promote AS into VEGF-Axxxb isoform [48]. Compounds blocking spliceosome machinery (Spliceostatin A, FR901464) [49, 50].
*VEGF receptors (VEGFRs)*	sVEGFR1	**Expression/function** Anti-angiogenic function, inhibits VEGF signalling in ECs [51]. Controversial role in cancer [52–54]. **Examples of potential use for therapy** Morpholino oligonucleotides to promote AS into sVEGFR1 [55].
	sVEGFR2	**Expression/function** Decreases lymphangiogenesis. Downregulated in neuroblastoma patients [56].
*Neuropilins (NRPs)*	sNRP1	**Expression/function** Soluble decoy receptor. Anti-angiogenic function [57–59]. **Examples of potential use for therapy** Overexpression of sNRP1 to prevent VEGF signalling [60].
	NRP1-∆7	**Expression/function** Altered glycosylation. Anti-angiogenic function [61].
	NRP1-∆E4, NRP1-∆E5	**Expression/function** Altered glycosylation and endocytic trafficking [62].
	s_9_NRP2	**Expression/function** Decoy function [63].
	Membrane-bound NRP2 variants	**Expression/function** Differentially activate signalling pathways [58].
*Fibroblasts growth factor receptors (FGFRs)*	FGFRIIIb	**Expression/function** Expressed by epithelial tissues [64]. Pro-angiogenic function [65, 66]. **Examples of potential use for therapy** Anti-FGFR2-IIIb–Specific Antibody (GP369) [67].
	FGFRIIIc	**Expression/function** Expressed in mesenchymal tissues [64] and primary ECs [68].
	sFGFRs	**Expression/function** Possible decoy function [69].
	Deletion of auto-inhibitory domain	**Expression/function** Hyper-activation of the signalling pathway [69].
	C-term FGFRs AS variants C1, C2, C3	**Expression/function** Differential impact on receptor internalization and downstream signalling. C3 implicated in oncogenesis [70].
	Deletion of VT motif	**Expression/function** Deletion affects downstream signalling [71].
*Vasohibins (VASHs)*	VASH1A	**Expression/function** Anti-angiogenic-function. Expressed by ECs [72]. **Examples of potential use for therapy** Overexpression of VASH1A [72].
	VASH1B	**Expression/function** Expressed by ECs. Promotes the normalization of tumor blood vessels [72]. **Examples of potential use for therapy** Overexpression of VASH1B [72].
	VASH2-355aa	**Expression/function** Expressed by ECs [73]; unknown function.
	VASH2-290aa	**Expression/function** Anti-angiogenic function [73].
*Angiopoietins (ANGs)*	ANG1–0.7, − 0.9 and − 1.3 kb	**Expression/function** Differentially activates TIE2 pathway [74].
	ANG2_443_	**Expression/function** Expressed in primary ECs and non-endothelial tumor cell lines. It antagonizes TIE2 signalling during tumorigenesis and inflammation [75].
	ANG2B	**Expression/function** Differentially activates TIE2 signalling [76].
*Fibronectin (FN)*	EDA/EDB-FN	**Expression/function** Expressed during embryonic and tumor angiogenesis. EDA-FN plays a role in vascular remodelling and prevents vascular oxidative stress in diabetic conditions [77, 78]. **Examples of potential use for therapy** Drug delivery [79].
*Tenascin C (TNC)*	Large TNC variants	**Expression/function** Expressed in pathological tissues undergoing active remodelling. Favour cell migration [80]. Specific spliced variants or single AS domains are associated with different tumor types [80] types; FNIII C-bearing TNC isoform is highly expressed in brain and lung tumors, associated with tumor stroma [81]. **Examples of potential use for therapy** TNC antibodies to deliver cytotoxic molecules, recognizing the AS domains A1 to D of the large isoform of TNC. Aptamer TTA1 [82].
*SLIT2*	Slit2-WT	**Expression/function** Expressed and released by tumor cells. Reduces EC permeability [83].
	Slit2-ΔE15	**Expression/function** Expressed and released by normal cells. Reduces EC permeability and plays a role in vessel normalization [83].
*PECAM1*	PECAM1-FL, Δ12, Δ13, Δ14, Δ15, Δ14&15	**Expression/function** PECAM1-FL is the major form of PECAM-1 in human tissues and ECs [84, 85]. Different isoforms bear different signalling potential, thus impacting angiogenesis process [86].
	sPECAM1	**Expression/function** Possible function in regulating PECAM1-mediated cellular interactions [87].
*CD146*	shCD146	**Expression/function** Promotes EC proliferation, migration and adhesion [88].
	lgCD146	**Expression/function** Promotes EC tube formation and stabilization [88].
*CD44*	CD44v6	**Expression/function** Controls EC migration, sprouting and tube formation, acting as a VEGFR2 co-receptor for VEGF-A [89]. **Examples of potential use for therapy** CD44v6 blockage by soluble peptides [90], humanized monoclonal antibody [91], shRNA [92], miRNA [93], or antisense oligonucleotides [94]. CAR-T cells against CD44v6+ cancer cells (ClinicalTrials.gov: NCT04427449 [95]).
*Endoglin (ENG)*	L-endoglin	**Expression/function** Interacts with TGFβ type I receptors ALK1, enhancing its-mediated pathway [96, 97].
	S-endoglin	**Expression/function** Interacts with TGFβ type I receptors ALK5, stimulating ALK5 pathway. Associated with altered pulmonary angiogenesis [98]. It is induced by senescence and able to contribute to NO-dependent vascular homeostasis.
*Insulin receptor (IR)*	IR-A	**Expression/function** Pro-proliferative function; overexpressed in tumor vasculature [99].
*Tissue factor (TF)*	asTF	**Expression/function** Soluble factor, highly expressed in advanced stages of several human cancers [100, 101]. Stimulates tumor growth, angiogenesis and metastasis [102]. **Examples of potential use for therapy** Antibody drug conjugate of TF and monomethyl auristatin E [103].
	flTF	**Expression/function** Highly expressed in several types of cancer. Involved in cancer-related thrombosis, tumor growth and metastasis [104]. **Examples of potential use for therapy** Anti-flTF antibody 10H10 [105].
*L1CAM (L1)*	L1-ΔTM	**Expression/function** Soluble form of L1CAM, released by ECs. Promotes EC tube formation and neovascularization. Overexpressed in the ovarian cancer vasculature; associated with tumor vascularization [106].
	L1-FL	**Expression/function** Highly expressed in tumor vasculature several types of cancer. Pro-angiogenic function [107].

#### VEGF-A

Among the growth factors, receptors, cytokines and enzymes controlling angiogenesis [108], VEGF-A is the main pro-angiogenic cytokine. It mainly binds its receptors (VEGFR1 and VEGFR2) exposed on EC surface [109].

AS regulation of *VEGFA* is paradigmatic. In particular, the differential usage of proximal and distal 3′ splice sites in exon 8 generates isoforms with different C-terminal domains and characterized by opposite properties, respectively being “pro-angiogenic” (VEGF-A_xxx_a, where xxx indicates the position of the amino acid residue in a specific isoform) or “anti-angiogenic” (VEGF-A_xxx_b) (Fig. 2a). These isoforms can also differ for their heparin-binding affinity, a region encoded by exons 6 and 7 [110]. While VEGF-A_xxx_b variants have not been detected in ECs, two pro-angiogenic AS variants are present in these cells, including VEGF-A_165_a, and VEGF-A_189_a (corresponding to the mouse proteins VEGF-A_164_a, and VEGF-A_188_a) [111]. Overexpression of these variants affects EC proliferation, adhesion, migration and the integrity of EC monolayers, as they all activate VEGFR2, although at a different extent [112]. Remarkably, VEGF-A_188_a is highly expressed in ECs from lung but not in tumor ECs, while VEGF-A_164_a increases in tumor versus normal ECs [47], in line with the pro-angiogenic phenotype of ECs in cancers.

Currently known *VEGFA* splicing regulators include members of SR protein family (i.e. SRSF1, SRSF2, SRSF5, and SRSF6) [113–115] and the serine-arginine protein kinase 1 (SRPK1) [116]. Phosphorylation of SRSF1 by SRPK1 determines SRSF1 nuclear localization that in turn promotes the usage of the proximal 3′ splice site and the production of the pro-angiogenic isoform VEGF-A_165_a [117]. Inhibition of SRPK1 reduces angiogenesis in vivo, setting it as a relevant target for anti-angiogenic therapy [48]. More recently, the circular RNA *circSMARCA5* has been identified as a sponge for SRSF1, controlling the ratio of VEGF-A pro- and anti-angiogenic isoforms in glioblastoma multiforme [118]. Moreover, SRSF2 and SRSF6, which both favor VEGF-A_xxx_b expression, are known to be regulated by the noncanonical WNT [119] and TGFβ1 pathways [46]. Finally, RBM10, an RBP modulated in cancer cells by epigenetic modifications of its promoter, has been associated with the production of the VEGF-A_165b_ anti-angiogenetic variant [120].

#### VEGF receptors (VEGFRs)

VEGFRs are tyrosine kinase receptors mediating VEGF signaling during both development and disease [121]. The family comprises three members, VEGFR1, VEGFR2 and VEGFR3, which exist as either membrane bound or soluble molecules, depending on AS. Soluble (s) isoforms of VEGFR1 (encoded by the *FLT1* gene) derive from the usage of alternative polyadenylation signals after partial retention of intron 13 (sVEGFR1-i13) or 14 (sVEGFR1-i14) or the terminal exons 15a and 15b (sVEGFR1-e15a/−e15b) [122]. All sVEGFR1 isoforms have an anti-angiogenic role, by either sequestering VEGF-A or forming inactive heterodimers with other VEGF receptors, thereby preventing downstream signaling [51].

The mechanisms leading to sVEGFR1 production in ECs are not fully elucidated. A role for hnRNP D has been described in HUVECs, in which its overexpression significantly decreases soluble/membrane-VEGFR1 ratio [123]. In addition, JMJD6 is involved in splicing regulation of *FLT1* [124], by interacting with the spliceosome component U2AF65, and thus stimulating the production of the membrane-bound isoform. Under hypoxic conditions, the interaction between JMJD6 and U2AF65 is inhibited and this generates the sVEGFR1-i13 variant [124]. A recent work suggests that the U2AF65/JMJD6 circuit could regulate the ECM enzyme heparanase to stimulate sVEGFR1 release from the ECM [125]. In cancer cells, VEGF-A_165_a cooperates with the transcription factor SOX2 and SRSF2 to promote sVEGFR1-i13 expression [126]. An additional layer of complexity is provided by the observation that VEGFR2 (encoded by the *KDR* gene) also exists in a soluble form (sVEGFR2), generated by the retention of a part of intron 13 [127]. By binding to VEGF-C, sVEGFR2 inhibits the activation of VEGFR3 during lymphatic EC proliferation [127].

#### Neuropilins (NRPs)

NRP1 and NRP2 are cell surface glycoproteins that act as co-receptors for different factors, such as VEGF and semaphorins [128]. NRP1 interacts with VEGFR1 or VEGFR2 in ECs, whereas NRP2 plays an important role in lymphangiogenesis thanks to its ability to dimerize with VEGFR3 [128]. *NRP1* pre-mRNA can be spliced in different isoforms. Some of these AS isoforms (s_11_NRP1, s_12_NRP1, s_III_NRP1, s_IV_NRP1), which lack the transmembrane domain (TM) and the cytoplasmic tail [57–59], are soluble proteins that act as decoy receptors by sequestering NRP1 ligands, thus exerting anti-angiogenic functions [57] (Fig. 2b). Another NRP1 splice variant (NRP1-∆7) derives from the usage of an alternative 5′ site in exon 11 leading to the deletion of 7 amino acids [61]. Such deletion impairs glycosylation of the NRP1-∆7 variant that fails to be internalized in the intracellular vesicles upon VEGF-A binding as well as to activate downstream pathways, thus acting as an anti-angiogenic protein [61]. More recently, other variants lacking exon 4 (NRP1-∆E4) or 5 (NRP1-∆E5) have been identified and characterized by altered glycosylation and endocytic trafficking, resulting in loss of cell migratory and invasive capacity [62].

NRP2 also exists as either membrane-bound or soluble isoforms, generated through AS. The soluble variant s_9_NRP2 results from intron 9 retention, which produces a truncated protein, exerting a decoy function by sequestering VEGF-C and inhibiting oncogenic VEGF-C/NRP2 signaling [63]. Membrane-bound NRP2 in turn exists in multiple AS forms, which differ in their cytosolic domain, suggesting diverse intracellular signaling pathways [58].

#### Fibroblasts growth factor receptors (FGFRs)

AS controls FGFR function at multiple levels [69]. For instance, the mutually exclusive usage of either exon 8 or exon 9 in *FGFR1–3* pre-mRNAs, encoding for the last portion of the immunoglobulin-like domain 3 (IgIII), generates the so called IIIb and IIIc isoforms, having different ligand specificity [129] (Fig. 2b). ECs mainly express the FGFR1IIIc, FGFR2IIIc, and FGFR3IIIc isoforms of FGFRs [68]. Intriguingly, an unbalance of FGFR-III splicing isoforms has been implicated in tumor angiogenesis and metastasis [130–133].

Among the RBPs influencing IIIb/IIIc isoform ratio are ESRP1, ESRP2, hnRNP F/H/K/M, RBM4, hnRNP A1, PTBP1, and PTBP2 [134–136]. An additional layer of complexity is also added by the epigenetic status of *FGFR1–3* genes, which can influence not only receptors expression [137], but also their isoform composition through splicing-specific histone modification patterns affecting the recruitment of PTB splicing factors [20].

Moreover, AS sustains the production of soluble variants through removal of the TM domain encoding exon [69]. Another AS event, resulting in the exclusion of exons encoding for FGFR auto-inhibitory domain, promotes the formation of hyper-activated receptors [69], whereas the inclusion of distinct C-terminal sequences in FGFR2 results in a differential composition in tyrosine residues, important for receptor phosphorylation [70]. Finally, exclusion of six nucleotides coding for the valine and threonine motif in the intracellular juxtamembrane region of FGFR1–3, impairs the binding of effector proteins, thus altering downstream signaling [71].

#### Vasohibins

Vasohibin-1 (VASH1) is an angiogenic inhibitor released by ECs in response to pro-angiogenic molecules [138]. Its AS produces two variants: *VASH1A* (full-length), and *VASH1B* (lacking exons 6–8) [72], which differ in their C-term domains (involved in heparin binding) and have opposite effects on ECs (Fig. 2b). While VASH1B inhibits angiogenesis, VASH1A promotes the normalization of tumor blood vessels [72], defined as the transient reduction (in structure and function) of the tumor vessels abnormalities. Vessel normalization is a novel concept in anti-angiogenesis targeting approaches. Indeed, by increasing blood perfusion and delivery of drugs, the normalization of the tumor vasculature could improve the responsiveness to chemotherapy, radiotherapy and immune cell therapy [139].

AS of *Vasohibin-2* (*VASH2)* generates multiple polypeptides of different length. In ECs, the full-length variant, composed of 355 amino acids, is the most represented, while another isoform of 290 amino acids exerts anti-angiogenic activity [73].

#### Angiopoietins

Angiopoietins (ANG1–4) are important modulators of vascular function by binding to TIE receptors. ANG1 is an agonist of TIE2, the activation of which promotes blood vessel stability, while ANG2 can act either as an antagonist or a weak agonist of TIE2, thereby regulating ANG1 activity with variable effects, depending on the context [140]. AS of *ANG1* gives rise to three shorter variants (0.7, 0.9 and 1.3 kb long), which show different capacity to phosphorylate TIE2 receptor, thereby regulating ANG1 function [74]. ANG2_443,_ generated by skipping of exon 2, binds the TIE2 receptor and it is expressed in primary ECs and in non-endothelial tumor cell lines [75]. This isoform, however, does not induce TIE2 phosphorylation and thus is an antagonist of TIE2 signaling during tumorigenesis and inflammation [75]. Finally, ANG2B, which derives from the inclusion of exon 1B, also modulates ANG2 activity and thus TIE2 signaling [76].

#### Fibronectin (FN)

FN, a component of the ECM, plays an important role in cell adhesion, migration, cell growth and differentiation [141]. The activity of FN is finely tuned by AS that mainly affects three FN regions: the extra domain A (EDA), the extra domain B (EDB), and the type III connecting sequence (IIICS) [77]. EDA- and EDB-containing isoforms (named oncofetal variants) are abundantly expressed during angiogenic conditions, such as embryogenesis and cancer [77]. In ECs, EDA-FN participates in vascular remodeling and prevents vascular oxidative stress in diabetic conditions [78]. Platelets and macrophages, recruited to the arterial endothelium, induce the expression of both EDA-FN and EDB-FN in response to change in blood flow [35]. In addition, the expression of EDA-FN and EDB-FN is induced in ECs by TGFβ in a SMAD3- and SMAD4-dependent manner, revealing an important interplay between TGFβ and FN signaling [142]. In ECs, SRSF5 and RBFOX2 mediate the expression of EDA-FN or EDB-FN [31, 143].

#### Tenascin C (TNC)

TNC is an extracellular matrix glycoprotein involved in cell adhesion and migration [80]. In glioma patients, TNC overexpression was correlated with vascular mimicry [144], the ability of cancer cells to create vascular channels independently by ECs [145]. Also in astrocytomas, TNC is upregulated specifically in ECs and not in tumor cells and its expression level correlates with angiogenic markers [146]. Several isoforms are generated through AS of exons encoding for fibronectin type III-like repeats (FNIII A1-D), in response to growth factors, inflammatory cytokines [80], and mechanical stresses [147]. Splicing isoforms of TNC are divided in “large” and “small”, depending on their molecular weight [80]. Whereas the smallest TNC isoform, lacking all AS FNIII exons, promotes cell adhesion, the larger TNC variants, generated by SRSF6 [148], favor cell migration [80]. Importantly, large TNCs are expressed in developing tissues and in pathological tissues that undergo active tissue remodeling, including tumors, pointing to these isoforms as promising targets in anti-cancer approaches [149]. Specific spliced variants or single AS domains have been associated with different kind of tumors [80]. In particular, the large TNC variant [80, 149], containing the FNIII C domain, is mainly expressed around vessels in high grade astrocytoma [81] but it is not present in normal tissues, suggesting that it could represent a therapeutic marker for this kind of tumor.

#### SLIT guidance ligand 2 (SLIT2)

SLIT2 is a secreted glycoprotein that binds the Roundabout (Robo) receptors and inhibits EC migration [150]. Depending on the context, it could have either pro- or anti-angiogenic effects [151]. In particular, secretion of SLIT2 by tumor cells generates a signaling gradient that attracts ECs as a fundamental step in the generation of a novel vessel network [152]. Skipping of exon 15 gives rise to the SLIT2-ΔE15 isoform. While SLIT2 full-length (FL) is expressed and released by tumor cells, SLIT2-ΔE15 is mainly present in normal tissues. Compared to the FL protein, SLIT2-ΔE15 reduces EC permeability and enhances tube formation [83].

#### PECAM1

PECAM1 is abundantly expressed in ECs, where it localizes at junctions and functions as regulator of vascular permeability [153]. The exons encoding the intracellular domain of PECAM1, which contains docking sites for signaling molecules, are subject to AS [154]. In particular, inclusion or exclusion of exons 12 to 15 leads to isoforms with peculiar roles in EC migration, adhesion, and tube formation [155, 156]. Through removal of the TM domain encoding exon, AS also generates a soluble form of PECAM1, which is able to inhibit adhesive interactions of the membrane-bound PECAM1 form [157].

#### CD146

CD146 has been recently proposed as a potential therapeutic target based on its involvement in vascular integrity [158]. Three forms of CD146 have been described and include two transmembrane isoforms, long CD146 (lgCD146) and short CD146 (shCD146), as well as a soluble isoform (sCD146), which circulates in the plasma and derives from metalloprotease-dependent shedding of the previous two proteins [158]. The lgCD146 and shCD146 isoforms are, respectively, generated by either inclusion or skipping of exon 15 and characterized by different intracellular domains, as well as by diverse cellular localization [158]. In ECs, lgCD146 is present at the junctions, whereas shCD146 localizes at the migrating front [88]. While shCD146 promotes EC proliferation, migration and adhesion, lgCD146 induces EC tube formation and stabilization [88].

#### CD44

CD44, a transmembrane glycoprotein involved in cell-cell and cell-matrix interactions, binds hyaluronic acid and other ECM components. A number of CD44 variants are generated through AS of 10 consecutive AS exons (v1 to 10) encoding for the extracellular juxtamembrane region. The short CD44 protein, lacking all alternative exons, is predominantly expressed in normal tissues, whereas CD44 variants containing exons v5, v6 and v7, are over-expressed in various cancers and associated to metastasis. In particular, the CD44v6 isoform controls EC migration, sprouting and tube formation through its ability to act as a VEGFR2 co-receptor for VEGF-A [89]. Blockage of co-receptor function of CD44v6 reduces tumor angiogenesis in vivo [89]. Moreover, AS is responsible for the production of a soluble variant of CD44 [159], which competes with membrane-bound CD44 protein on EC surface.

#### Endoglin (ENG)

ENG, an auxiliary receptor for TGFβ, is mainly expressed on proliferating ECs and upregulated during hypoxia [160]. A short isoform of endoglin (S-endoglin) results from the retention of intron 13. The canonical long (L-endoglin) and the short S-endoglin proteins differ in their cytoplasmic tails and for their ability to interact with TGFβ type I receptors ALK1 and ALK5 (Fig. 2b). L-endoglin enhances ALK1 signaling, while S-endoglin promotes ALK5 activation [96, 97]. S-endoglin expression is induced in ECs during senescence and is involved in NO-dependent vascular homeostasis. In senescent ECs, SRSF1 leads to an increased expression of *S-endoglin* mRNA [161]. More recently, S-endoglin-mediated ALK5 signaling has been related to altered pulmonary angiogenesis induced by hyperoxia [98].

#### Insulin receptor (IR)

IR (encoded by *INSR*) has been proposed as tumor EC marker, as it is overexpressed by the vasculature of different cancer types, but not by activated endothelium in physiological conditions [99]. In addition, increased expression of vascular IR is correlated with bad prognosis of cancer patients. AS of *INSR* gives rise to two different variants: IR-A and IR-B. These two isoforms differ in ligand affinity and cellular downstream signaling [162]. In particular, IR-B is the full-length protein mediating the metabolic function of IR, while the shorter IR-A (lacking exon 11) controls cell proliferation [99]. Since IR-A is overexpressed by the tumor vasculature [99] it could represent a potential target for anti-angiogenic therapies.

#### Tissue factor (TF)

TF is a cell surface glycoprotein involved in vessel formation and maturation, as well as in the activation of blood clotting cascade. TF undergoes AS to generate multiple isoforms. In particular, skipping of exon 5 generates the soluble factor asTF (alternatively spliced TF) [163], which lacks any pro-coagulant activity, stimulates tumor growth, angiogenesis, and metastasis [102]. Its expression levels positively correlate with progression in several cancers [100, 101].

#### Cell adhesion molecule L1 (L1CAM)

L1CAM orchestrates important EC functions, in particular in tumor vasculature [106]. An EC-specific variant of L1CAM (L1-ΔTM) is generated through skipping of exon 25, which removes the TM domain and generates a soluble protein [106] (Fig. 2b). In ECs, the splicing regulator NOVA2 stimulates L1-ΔTM production through direct binding to RNA motifs in exon 25. L1-ΔTM promotes EC tube formation and sustains neovascularization in vivo in a FGFR1-dependent manner. L1-ΔTM is overexpressed in the vasculature of ovarian cancer, where its expression levels correlate with tumor vascularization [106].

### SRFs regulating EC functions

A list of SRFs relevant for vascular development is shown in Supplementary Table 2 (Additional files 1 and 2), based on the Mouse Genome Information (MGI) [164] and the Zebrafish Information Network (ZFIN) [165], which provide information on mouse gene and zebrafish knockouts and their phenotypes. Here, we discuss the current knowledge on SRF critically involved in ECs biology.

#### PTBP1

PTBP1, a broadly expressed SRF, coordinates AS in a variety of processes, including acquisition of cellular morphology, immunity, metabolic control and cell cycle [166]. PTBP1 is expressed at high levels in ECs of different tissues and its expression levels correlate with the inclusion rate of EC-specific exons, particularly in genes involved in cell-cell or cell–matrix adhesion [167].

Quiescent ECs express low levels of PTBP1 [168], while its expression increases in pathological conditions. In pulmonary hypertension, PTBP1 levels increase in arterial vessels, due to partial loss of its negative regulator *miR-124 *[168]. *PTBP1* is also upregulated in blood vessels of glioblastoma multiforme, one of the most aggressive brain cancers [169], and glioma, in which its depletion significantly increases blood-tumor barrier permeability [170]. Consistent with the pro-angiogenic activity of PTBP1, medium conditioned by PTBP1-knockdown cells lost the capacity to promote tube formation by HUVECs [171, 172].

#### SRSF1

SRSF1 is involved in different aspects of RNA metabolism, including splicing, mRNA stability, translation, and miRNA processing [173]. SRSF1 is frequently upregulated in different cancers [24] and a direct target of the oncogenic transcription factor c-Myc [174]. SRSF1 overexpression is sufficient to immortalize rodent fibroblasts and form tumors in mice [24, 175], whereas its depletion promotes genomic instability, apoptosis and cell-cycle arrest [176, 177]. AS regulated by SRSF1 generates protein variants involved in cell migration, epithelial to mesenchymal transition [178], oncogenic activation, loss of tumor suppressor activity [24, 179, 180] and angiogenesis [181].

SRSF1 controls EC senescence [161] and their response to vascular injury [182]. While it is barely expressed in normal ECs, it increases in cancer ECs [47], often accompanied by upregulation of the pro-angiogenic VEGF-A_164_a isoform [47] and associated to increased microvessel density [118].

Endothelial SRSF1 expression is induced by the Wilm’s tumor suppressor 1 (WT1) transcription factor, whereas its activity is regulated by SRPK, which favors SRSF1 nuclear localization [181]. Knockout of WT1 in tumor endothelium decreased SRPK1 and SRSF1 expression and shifted *VEGFA* splicing toward the production of the anti-angiogenic *VEGF-A*_*120*_ isoform [47].

#### NOVA2

Initially considered neuronal-specific [183], NOVA2 is actually expressed by ECs in different blood vessels [184]. For instance, it is abundant in mouse cardiac ECs [185] and preferentially expressed by veins compared to arteries in zebrafish [186]. NOVA2 depletion in ECs impairs the acquisition of cell polarity and the organization of cell-cell junctions, resulting in increased EC migration and permeability [184]. Consistently, *nova2* zebrafish mutants present many vascular defects [184]. NOVA2 modulates AS of genes involved in EC cytoskeleton organization and cell-cell adhesion, as well as the transcription factors PPAR-γ and E2F Dimerization Partner 2 (Tfdp2) [187]. Very recently NOVA2 was shown to modulate AS of components of Mapk/Erk pathway during lymphatic EC specification [186]. In cancer, such as ovarian and colorectal carcinomas, NOVA2 expression is specifically upregulated in tumor ECs [106, 188] and correlates with low survival [106], supporting its potential role as a prognostic marker. A positive correlation between NOVA2 and HIF1-α was observed in colorectal cancer [188], consistent with upregulation of NOVA2 in HUVECs cultured in hypoxic conditions [188].

#### MBNLs

MBNLs are tissue-specific RBPs. While MBNL1 is ubiquitously expressed, MBNL2 and MBNL3 are essentially confined to brain and muscle, respectively [189]. MBNL1/2 are upregulated in mature ECs compared to their progenitors [190]. MBNL2 expression has also been reported in HUVECs [191], whereas MBNL1 was found to be expressed and mislocalized in corneal ECs during pathological condition [192]. Several MBNL1-regulated genes are involved in angiogenesis (i.e. *VEGFA, ADD3, INF2, SORBS1, FGFR1),* EMT, Rho-mediated cytoskeleton dynamics (*ARHGEF40, AKAP16*) and cell-cell junctions (*PPHLN1*) [192].

#### ELAVL1

ELAVL1, which is involved in a number of physiological processes (i.e. cell proliferation, differentiation, apoptosis) as well as pathologic conditions (i.e. cancer and inflammation) [193], has been mainly characterized for its ability to stabilize mRNA targets. However, it also acts as a SRF [193]. Endothelial-specific knockout of ELAVL1 does not impair either embryonic vascular development or postnatal angiogenesis in adult mice [194]. Nevertheless, these mice are characterized by reduced re-vascularization after hind limb ischemia as well as decreased tumor angiogenesis [194]. In addition, *ELAVL* knockdown zebrafish embryos show aberrant vascular structures in the intestinal plexus [195]. Consistently, loss of ELAVL1 in cultured ECs impairs their migration and sprouting [194]. Among ELAVL1 splicing targets, *Eukaryotic translation initiation factor 4E nuclear import factor 1* (*EIF4ENIF1*) [194] encodes for the translation initiation factor 4E transporter (4E-T). Depletion of ELAVL1 causes the production of a short isoform (4E-Ts) that accelerates degradation of angiogenic regulatory mRNAs (*FOS, HIF1-α, VEGFA*). ELAVL1 is localized in the cytoplasm of tumor ECs, in which it controls survival, migration and tube formation [196].

#### RBFOX2

RBFOX proteins (RBFOX1, RBFOX2 and RBFOX3) control AS in brain [197]. However, RBFOX2 is also expressed by the arterial ECs, in which it mediates the cellular response to low blood flow [31]. A number of EC-specific *RBFOX2* splicing targets encode for ECM components or factors involved in cell adhesion, cell cycle, cytoskeletal remodeling and immune response [31]. Similar to NOVA2 [184, 187], RBFOX2 also regulates the abundance of mRNAs transcribed from genes that belong to the same GO categories [31], suggesting that similar biological processes could be modulated by RBFOX2 in ECs through both transcriptional and post-transcriptional mechanisms.

### Therapeutic strategies exploiting AS of angiogenic factors in cancer

Since multiple alterations in AS appear to be specific for cancer angiogenesis, the obvious implication is whether we can manipulate and therapeutically block this process, hence disfavoring tumor growth.

Multiple molecular tools have been exploited to target aberrant AS variants (Table 2). The most common ones are monoclonal antibodies, small molecules, and various types of oligonucleotides. These include: i) small interfering RNAs (siRNAs) targeting one particular AS isoform, ii) modified single stranded RNA decoy oligonucleotides inhibiting the biological activity of splicing regulators, and iii) splicing-switching oligos, ~ 20 base long modified oligonucleotides binding specific splicing regulatory sites.

**Table 2:** Therapeutic strategies (Pros & Cons)

Therapeutic approach	Examples	Pros and cons
**Controlling the activity of splicing factor regulators**	- Small molecules targeting SRPK1 (SPHINX, SRPIN340 and SRPKIN-1) used for *VEGFA* splicing correction [48, 198].	Poor specificity, resulting in AS modification of multiple genes besides *VEGFA*.
**Inhibiting the assembly of the spliceosome machinery**	- Compounds binding to the spliceosome component SF3b: FR901464 and its methylated derivative, spliceostatin A [199].	Poor specificity, affecting AS of multiple genes; partial understanding of mechanism of action.
**Interfering with splicing sites**	- Morpholino oligonucleotides targeting the exon 13/intron 13 junction of the *VEGFR1* pre-mRNA, favoring the production of the anti-angiogenic, soluble form of VEGFR1 [55].	Possibility to target one single gene; off-target effects due to either the presence of the targeted sequence in other portions of the genome or tolerance toward mismatches.
**Blocking pro-angiogenic isoforms**	- Humanized monoclonal antibody [91] or a soluble peptide [200, 201] against CD44v6. - Intravenous delivery of autologous T cells, modified to recognize CD44v6 on the surface of cancer cells (ClinicalTrials.gov: NCT04427449 [95]). - Monoclonal antibodies against FGF8b [202]; using natural inhibitor Pentraxin-3 (PTX3) and its derivatives Ac-ARPCA-NH2 (ARPCA) and 8b-13 [203, 204] to target FGFs.	High specificity with minimal side effects; cumbersome and expensive design and production.
**Overexpressing anti-angiogenic isoforms**	- Overexpression of sNRP1 to prevent VEGF signalling [60]. - Overexpression of either VASH1B or VASH1A [72].	Delivery requiring either gene therapy or production of recombinant proteins; no effect on the level of pro-angiogenic isoforms.
**Exploiting cancer-specific isoforms for drug delivery**	- Monoclonal antibodies and aptides targeting EDA/EDB domains of FN: F8 fused to IL-2 [205, 206]; L19 fused to either IL-2 or IL-12 [207, 208]; EDB-targeting aptides conjugated with doxorubicin-containing liposomes [209, 210]. - Monoclonal antibodies (F16 fused to IL-2) and aptamers targeting domains A1-D of TNC [211].	High specificity for cancer cells; cumbersome and expensive design and production; toxicity related to the chemotherapeutic agent.

These tools have been variably used to interfere with cancer-specific AS. The following paragraphs describe the strategies that have been so far considered most promising for human application. An overview of the existing approaches, together with their major advantages and disadvantages, is provided in Table 2.

#### Drugs targeting splicing factor regulators

SRPK1 activity has been associated to increased tumor cell proliferation, migration and angiogenesis in different cancers [212, 213]. The evidence that SRPK1 inhibition switches the pro-angiogenic VEGF-A_165_a into the anti-angiogenic VEGF-A_165_b isoform [181] leaded to the generation of a plethora of small molecules targeting SRPK1, such as SPHINX and its derivatives, SRPIN340 and SRPKIN-1, which are the most effective ones in correcting *VEGFA* splicing. These molecules are able to efficiently block angiogenesis in murine models of both macular degeneration and cancer [48, 198].

#### Inhibitors of spliceosome assembly

One of the first approaches able to interfere with AS in cancer angiogenesis exploits compounds inhibiting the spliceosome assembly. A paradigmatic example is the natural product FR901464 and its methylated derivative, spliceostatin A, which binds to the spliceosome component SF3b [199]. In a chicken chorioallantoic membrane (CAM) assay, spliceostatin A reduced the expression of 38% of total genes (including *VEGFA*) and inhibited cancer cell-derived angiogenesis [49].

#### Interference with splicing sites

Chemically modified antisense oligonucleotides, targeting sequences recognized by the spliceosome or splicing factors, can be exploited to re-direct splice site selection and to correct AS decisions. While their use is widely exploited to interfere with a variety of molecules controlling cancer cell survival and proliferation [214], a few studies have started investigating their therapeutic potential in modulating cancer angiogenesis. Interestingly, morpholino oligonucleotides targeting the exon 13/intron 13 junction of the *VEGFR1* pre-mRNA, have been used to favor the production of the anti-angiogenic, soluble form of the receptor (sVEGFR1). The repeated injection of these oligonucleotides in human breast cancer tumors, implanted subcutaneously into nude mice, inhibited cancer vascularization and progression [55].

#### Blocking pro-angiogenic splicing isoforms

An obvious approach to modulate AS in cancer angiogenesis is the selective inhibition of pro-angiogenic isoforms. This can be efficiently achieved using peptides, monoclonal antibodies or chimeric antigen receptor (CAR)-T cells. Numerous experimental and clinical studies are targeting pro-angiogenic isoforms of CD44, which are expressed by multiple cancer cell types. Current strategies mainly target CD44v6, using either a humanized monoclonal antibody [91] or a soluble peptide [200, 201, 215] that blocks exon v6-encoded domain. A clinical trial is currently ongoing to evaluate the efficacy of the intravenous delivery of autologous T cells, genetically modified with lentiviral CAR vector, to recognize CD44v6 on the surface of cancer cells (ClinicalTrials.gov: NCT04427449 [95]). Additional strategies, which have not been tested in human cancer, target FGF ligands, with particular attention to some FGF isoforms that are preferentially expressed by specific tumor types. For example, the activity of FGF8b, overexpressed by hormone-dependent tumors, can be blocked using either monoclonal antibodies [202] or its natural inhibitor Pentraxin-3 (PTX3) and its derivatives Ac-ARPCA-NH2 (ARPCA) and 8b-13. While these peptides also block FGF2, they show higher affinity for FGF8b. In particular, FGF8b inhibition by ARPCA decreased HUVECs migration and sprouting, and resulted in reduced proliferation and vascularization of androgen-dependent mouse mammary tumors implanted into the flank of nude mice [203, 204].

#### Overexpression of (naturally existing) anti-angiogenic splicing isoforms

Anti-angiogenic isoforms can be overexpressed to block tumor vascularization. Starting from the evidence that soluble neuropilins prevent VEGF signalling, sNRP1 has been overexpressed by adenoviral vectors, resulting in reduced angiogenesis and delayed disease progression in mouse models of myeloid sarcoma and acute myeloid leukemia [60].

An additional example in this category is the overexpression of either VASH1B, which induced tumor necrosis in murine model of human breast carcinoma, or VASH1A, which resulted in tumor vessel normalization and improved perfusion. The simultaneous overexpression of both isoforms was even more effective in inhibiting cancer growth and normalizing its vasculature [72].

#### Targeting cancer-specific AS isoforms for drug delivery

The evidence that the tumor vasculature tends to selectively express specific AS isoforms paved the way to target them to facilitate drug delivery to the neoplastic mass.

Several compounds and peptides have been developed to target either the EDA or the EDB domains of fibronectin [79]. For instance, the F8 monoclonal antibody, targeting EDA, has been fused to IL-2 to stimulate the immune system specifically at the level of the tumor. This strategy successfully inhibited the tumor growth in multiple models of murine xenografts, particularly when associated to either chemotherapeutic drugs or anti-angiogenic molecules [205, 206]. A similar strategy has been used even more widely to target EDB. The human EDB domain specific antibody, L19 was particularly effective in both pre-clinical and clinical studies, when fused to either IL-2 or IL-12 [207, 208].

In addition to antibodies, peptides have been generated to target fibronectin for tumor drug delivery. Aptides are short high-affinity peptides consisting of two EDB-targeting moieties linked by a tryptophan zipper region. When conjugated with doxorubicin-containing liposomes, they promoted drug delivery to glioma tumor allografts in mice, determining a 55% decrease in tumor size compared to 20% decrease induced by free doxorubicin [209, 210].

Finally, the preferential expression of long TNC isoforms in cancer can also be targeted for drug delivery. Antibodies targeting the AS domains A1 to D (variably present in the longer isoforms of TNC) [216] have been evaluated in preclinical studies and a few have reached the clinical arena. The most advanced results are available for one of these antibodies (F16) fused to IL2 for the therapy of different metastatic cancers [211]. The same TNC domains can be targeted using aptamers, which can be chemically synthetized and, being small molecules, show superior biodistribution compared to monoclonal antibodies. The specificity of these aptamers (i.e. TTA1 and GBI-10) has been proven in vitro, but their in vivo application has never been tested yet.

## Conclusions

Based on its pervasive use and its high molecular versatility, AS has a central role in gene expression regulation in human cells. However, unlike the well-characterized pathways controlling angiogenesis at transcriptional level, our knowledge of how AS impacts on EC functions are still limited. Thus, future works are needed to i) characterize the functional role of most AS variants in ECs; ii) better understand how *cis*-acting motifs and their cognate RBPs act together to modulate AS of specific genes, and iii) comprehend how the splicing is integrated with other cellular processes (such as transcription, epigenetic modifications and signaling pathways).

In cancer vessels tumor ECs express several atypical splicing isoforms not expressed (or expressed at low levels) in normal ECs, which could represent putative targets for anti-angiogenic therapy. Indeed, aberrant AS in tumor vasculature is emerging as a promising concept paving the way to anti-cancer therapeutic strategies. A deeper understanding of the AS errors occurring during cancer development and progression could allow formulating more specific and effective therapies. To what extent AS is specifically altered in different tumor types remains an outstanding question. The answer will possibly set AS in the field of theranostics, a new medical area combining targeted therapies with specifically targeted diagnostic tests. Since AS can be interrogated by common and relatively inexpensive techniques (i.e. RT-PCR), it could be rapidly analyzed at the time of tumor resection to select the most effective combination of drugs for each patient. Among the different strategies considered so far, monoclonal antibodies represent perhaps the most promising approach, as they are already in clinical practice for numerous disorders, including cancer, and platforms for their production, albeit expensive, could be easily adapted to new use. The possibility to fuse them to immune regulators, triggering patient’s immune response directly at the tumor site, further extends their therapeutic potential. Finally, the emerging evidence of the existence of cancer-specific AS isoforms will surely offer new opportunities for combination therapies, as standard chemotherapy can be potentiated by targeting these AS isoforms to induce vessel normalization, thus improving perfusion and drug delivery.

Understanding the contribution of AS regulation in tumor angiogenesis goes beyond the possibility of directly exploiting it as a source of new therapeutic targets. Indeed, identifying AS variants in cancer vasculature - as well as studying their functions and the molecular mechanisms underlying their production - would deepen our comprehension of the angiogenic process and allow to discover novel pathways relevant for cancer progression.

## Supplementary Information

**Additional file 1:** **Supplementary Table 1.** Additional AS isoforms (or events) relevant for angiogenesis and EC biology. **Supplementary Table 2.** RBPs whose deficiency results in aberrant vascular phenotypes in mice (MGI database) or Zebrafish (*Danio rerio)* (ZFIN database).

**Additional file 2.** Additional References.

## Data Availability

N/A.

## Abbreviations

AS: Alternative Splicing
EC(s): Endothelial Cell(s)
Pre-mRNA: Precursor messenger RNA
RBPs: RNA Binding Proteins
SR: Serine-Arginine rich
hnRNPs: Heterogeneous Nuclear Ribonucleoproteins
SRFs: Splicing Regulatory Factors
RNA pol II: RNA Polymerase II
RNA-seq: RNA Sequencing
ECM: Extracellular Matrix
HUVECs: Human Umbilical Vein Endothelial Cells
TM: Transmembrane Domain
FL: Full Lenght
CAM: Chorioallantoic Membrane
CAR: Chimeric Antigen Receptor
PTX3: Pentraxin 3
RT-PCR: Reverse Transcription-Polymerase Chain Reaction
ISS: Intronic Splicing Silencer
ESS: Exonic Splicing Silencer
ESE: Exonic Splicing Enhancer
ISE: Intronic Splicing Enhancer
BP: Branch Point
SSOs: Splice-Switching Oligonucleotides
