# Supplementary material for: Alternative splicing in endothelial cells: novel therapeutic opportunities in cancer angiogenesis
Source: J Exp Clin Cancer Res. 2020 Dec 7;39:275. doi: 10.1186/s13046-020-01753-1 (PMC7720527; doi:10.1186/s13046-020-01753-1)
Supplement: Supplementary file 2 — Additional file 2. Additional References. [file 13046_2020_1753_MOESM2_ESM.docx]

**Additional References**

1. Mollet IG, Ben-Dov C, Felício-Silva D, Grosso AR, Eleutério P, Alves R, et al. Unconstrained mining of transcript data reveals increased alternative splicing complexity in the human transcriptome. Nucleic Acids Res. 2010;38:4740–54.

2. ExonMine. https://imm.medicina.ulisboa.pt/group/exonmine/. Accessed 19 Jul 2020.

3. Kemmerer K, Weigand JE. Hypoxia reduces MAX expression in endothelial cells by unproductive splicing. FEBS Lett. 2014;588:4784–90.

4. Appukuttan B, McFarland TJ, Davies MH, Atchaneeyasakul L, Zhang Y, Babra B, et al. Identification of novel alternatively spliced isoforms of RTEF-1 within human ocular vascular endothelial cells and murine retina. Invest Ophthalmol Vis Sci. 2007;48:3775–82.

5. Hang X, Li P, Li Z, Qu W, Yu Y, Li H, et al. Transcription and splicing regulation in human umbilical vein endothelial cells under hypoxic stress conditions by exon array. BMC Genomics. 2009;10:126.

6. Sun L, Vitolo MI, Qiao M, Anglin IE, Passaniti A. Regulation of TGFβ1-mediated growth inhibition and apoptosis by RUNX2 isoforms in endothelial cells. Oncogene. 2004;23:4722–34.

7. Gaffney CJ, Oka T, Mazack V, Hilman D, Gat U, Muramatsu T, et al. Identification, basic characterization and evolutionary analysis of differentially spliced mRNA isoforms of human YAP1 gene. Gene. 2012;509:215–22.

8. Wu X, Pan W, Stone KP, Zhang Y, Hsuchou H, Kastin AJ. Expression and signaling of novel IL15Ralpha splicing variants in cerebral endothelial cells of the blood-brain barrier. J Neurochem. 2010;114:122–9.

9. Molina E, Hermida J, López-Sagaseta J, Puy C, Montes R. The functional properties of a truncated form of endothelial cell protein C receptor generated by alternative splicing. Haematologica. 2008;93:878–84.

10. Lasagni L, Francalanci M, Annunziato F, Lazzeri E, Giannini S, Cosmi L, et al. An alternatively spliced variant of CXCR3 mediates the inhibition of endothelial cell growth induced by IP-10, Mig, and I-TAC, and acts as functional receptor for platelet factor 4. J Exp Med. 2003;197:1537–49.

11. Heusschen R, Schulkens IA, van Beijnum J, Griffioen AW, Thijssen VL. Endothelial LGALS9 splice variant expression in endothelial cell biology and angiogenesis. Biochim Biophys Acta. 2014;1842:284–92.

12. Britto JM, Lukehurst S, Weller R, Fraser C, Qiu Y, Hertzog P, et al. Generation and characterization of neuregulin-2-deficient mice. Mol Cell Biol. 2004;24:8221–6.

13. Nakano N, Higashiyama S, Ohmoto H, Ishiguro H, Taniguchi N, Wada Y. The N-terminal region of NTAK/neuregulin-2 isoforms has an inhibitory activity on angiogenesis. J Biol Chem. 2004;279:11465–70.

14. Yu L, Cecil J, Peng S-B, Schrementi J, Kovacevic S, Paul D, et al. Identification and expression of novel isoforms of human stromal cell-derived factor 1. Gene. 2006;374:174–9.

15. Chang C-W, Seibel AJ, Avendano A, Cortes-Medina MG, Song JW. Distinguishing Specific CXCL12 Isoforms on Their Angiogenesis and Vascular Permeability Promoting Properties. Adv Healthc Mater. 2020;9:e1901399.

16. Döring Y, van der Vorst EPC, Duchene J, Jansen Y, Gencer S, Bidzhekov K, et al. CXCL12 Derived From Endothelial Cells Promotes Atherosclerosis to Drive Coronary Artery Disease. Circulation. 2019;139:1338–40.

17. Ho TK, Tsui J, Xu S, Leoni P, Abraham DJ, Baker DM. Angiogenic effects of stromal cell-derived factor-1 (SDF-1/CXCL12) variants in vitro and the in vivo expressions of CXCL12 variants and CXCR4 in human critical leg ischemia. J Vasc Surg. 2010;51:689–99.

18. Rust R, Grönnert L, Gantner C, Enzler A, Mulders G, Weber RZ, et al. Nogo-A targeted therapy promotes vascular repair and functional recovery following stroke. Proc Natl Acad Sci U S A. 2019;116:14270–9.

19. Cai H, Saiyin H, Liu X, Han D, Ji G, Qin B, et al. Nogo-B promotes tumor angiogenesis and provides a potential therapeutic target in hepatocellular carcinoma. Mol Oncol. 2018;12:2042–54.

20. Retta SF, Avolio M, Francalanci F, Procida S, Balzac F, Degani S, et al. Identification of Krit1B: a novel alternative splicing isoform of cerebral cavernous malformation gene-1. Gene. 2004;325:63–78.

21. Jiang X, Padarti A, Qu Y, Sheng S, Abou-Fadel J, Badr A, et al. Alternatively spliced isoforms reveal a novel type of PTB domain in CCM2 protein. Sci Rep. 2019;9:15808.

22. Hueso M, Cruzado JM, Torras J, Navarro E. An exonic switch regulates differential accession of microRNAs to the Cd34 transcript in atherosclerosis progression. Genes. 2019;10:70.

23. Cybulsky MI, Fries JW, Williams AJ, Sultan P, Eddy R, Byers M, et al. Gene structure, chromosomal location, and basis for alternative mRNA splicing of the human VCAM1 gene. Proc Natl Acad Sci U S A. 1991;88:7859–63.

24. Mascarenhas JB, Tchourbanov AY, Danilov SM, Zhou T, Wang T, Garcia JGN. The splicing factor hnRNPA1 regulates alternate splicing of the MYLK Gene. Am J Respir Cell Mol Biol. 2018;58:604–13.

25. Sugimura K, Tian XL, Hoffmann S, Ganten D, Bader M. Alternative splicing of the mRNA coding for the human endothelial angiotensin-converting enzyme: a new mechanism for solubilization. Biochem Biophys Res Commun. 1998;247:466–72.

26. Rashed L, Hay RA, Mahmoud R, Hasan N, Zahra A, Fayez S. Association of angiotensin-converting enzyme (ACE) gene polymorphism with inflammation and cellular cytotoxicity in vitiligo patients. PLoS One. 2015;10:e0132915.

27. Komatsu T, Suzuki Y, Imai J, Sugano S, Hida M, Tanigami A, et al. Molecular cloning, mRNA expression and chromosomal localization of mouse angiotensin-converting enzyme-related carboxypeptidase (mACE2). DNA Seq. 2002;13:217–20.

28. Wakasugi K, Slike BM, Hood J, Otani A, Ewalt KL, Friedlander M, et al. A human aminoacyl-tRNA synthetase as a regulator of angiogenesis. Proc Natl Acad Sci U S A. 99:173–7.

29. Eisenreich A. Regulation of vascular function on posttranscriptional level. Thrombosis. 2013;2013:948765.

30. Azoitei N, Becher A, Steinestel K, Rouhi A, Diepold K, Genze F, et al. PKM2 promotes tumor angiogenesis by regulating HIF-1α through NF-κB activation. Mol Cancer. 2016;15:3.

31. Stone OA, El-Brolosy M, Wilhelm K, Liu X, Romão AM, Grillo E, et al. Loss of pyruvate kinase M2 limits growth and triggers innate immune signaling in endothelial cells. Nat Commun. 2018;9:4077.

32. Mohun T, Adams DJ, Baldock R, Bhattacharya S, Copp AJ, Hemberger M, et al. Deciphering the mechanisms of developmental disorders (DMDD): a new programme for phenotyping embryonic lethal mice. Dis Model Mech. 2013;6:562–6.

33. Zhang J, Modi Y, Yarovinsky T, Yu J, Collinge M, Kyriakides T, et al. Macrophage β2 integrin-mediated, HuR-dependent stabilization of angiogenic factor-encoding mRNAs in inflammatory angiogenesis. Am J Pathol. 2012;180:1751–60.

34. Dickinson ME, Flenniken AM, Ji X, Teboul L, Wong MD, White JK, et al. High-throughput discovery of novel developmental phenotypes. Nature. 2016;537:508–14.

35. IMPC | International Mouse Phenotyping Consortium. https://www.mousephenotype.org/. Accessed 19 Jul 2020.

36. Bollmann F, Wu Z, Oelze M, Siuda D, Xia N, Henke J, et al. Endothelial dysfunction in tristetraprolin-deficient mice is not caused by enhanced tumor necrosis factor-α expression. J Biol Chem. 2014;289:15653–65.

37. Ghanem LR, Kromer A, Silverman IM, Chatterji P, Traxler E, Penzo-Mendez A, et al. The poly(C) binding protein Pcbp2 and its retrotransposed derivative Pcbp1 are independently essential to mouse development. Mol Cell Biol. 2016;36:304–19.

38. Lu JY, Sadri N, Schneider RJ. Endotoxic shock in AUF1 knockout mice mediated by failure to degrade proinflammatory cytokine mRNAs. Genes Dev. 2006;20:3174–84.

39. Lu ZH, Books JT, Ley TJ. YB-1 is important for late-stage embryonic development, optimal cellular stress responses, and the prevention of premature senescence. Mol Cell Biol. 2005;25:4625–37.

40. Bohnsack BL, Lai L, Northrop JL, Justice MJ, Hirschi KK. Visceral endoderm function is regulated by quaking and required for vascular development. Genesis. 2006;44:93–104.

41. Sen S, Jumaa H, Webster NJG. Splicing factor SRSF3 is crucial for hepatocyte differentiation and metabolic function. Nat Commun. 2013;4:1336.

42. Mende Y, Jakubik M, Riessland M, Schoenen F, Rossbach K, Kleinridders A, et al. Deficiency of the splicing factor Sfrs10 results in early embryonic lethality in mice and has no impact on full-length SMN/Smn splicing. Hum Mol Genet. 2010;19:2154–67.

43. Zhou W, Chung YJ, Parrilla Castellar ER, Zheng Y, Chung HJ, Bandle R, et al. Far upstream element binding protein plays a crucial role in embryonic development, hematopoiesis, and stabilizing myc expression levels. Am J Pathol. 2016;186:701–15.

44. Sakakibara S, Nakamura Y, Yoshida T, Shibata S, Koike M, Takano H, et al. RNA-binding protein Musashi family: roles for CNS stem cells and a subpopulation of ependymal cells revealed by targeted disruption and antisense ablation. Proc Natl Acad Sci U S A. 2002;99:15194–9.

45. Maragh S, Miller RA, Bessling SL, McGaughey DM, Wessels MW, de Graaf B, et al. Identification of RNA binding motif proteins essential for cardiovascular development. BMC Dev Biol. 2011;11:62.
